# Modeling and analysis of transient lubrication characteristic of the helicopter main transmission spiral bevel gears in point contacts

**DOI:** 10.1038/s41598-021-00284-9

**Published:** 2021-10-22

**Authors:** Yanzhong Wang, Kai Yang, Xiaomeng Chu, Wen Tang, Changyong Huang

**Affiliations:** 1 School of Mechanical Engineering, Beihang University, Beijing, 100191 China; 2 College of Mechanical Engineering and Automation, Liaoning University of Technology, Jinzhou, 121001 China; 3 Ningbo Institute of Technology, Beihang University, Ningbo, 315800 China; 4 China Electronics Technology Group Corporation 3rd Research Institute, Beijing, 100015 China

**Keywords:** Energy science and technology, Engineering

## Abstract

An engineering calculation model is introduced for point-contact elastohydrodynamic lubrication analysis of spiral bevel gears. This model can analyze transient lubrication characteristics of spiral bevel gears. The influence of the angle between the lubricant entrainment and the minor axis of the contact ellipse is included in this model. The contact parameters of the spiral bevel gear are calculated, which will change with time during the meshing process. The variation of lubricant film thickness during the meshing process of spiral bevel gears is unraveled. Due to the influence of entrainment velocity, the oil film thickness at the out mesh side is smaller than that at the enter mesh side under the same contact force. It is evident that the higher the pressure is, the larger the contact area will be. Meanwhile, the thickness of the oil film is reduced, and the oil film distribution in the contact area is relatively uniform. Taking helicopter main transmission spiral bevel gears as an example, this study finally calculates the distribution characteristics of the oil film thickness of the spiral bevel gear, and solves the lubrication performance of the spiral bevel gear under different working conditions.

## Introduction

The elastohydrodynamic lubrication (EHL) can be applied to the study of common engineering contact elastohydrodynamic lubrication areas such as gear meshing and rolling bearings. For helicopter main transmission spiral bevel gears, high input speed and high slip speed lead to high heating of gear system. At the same time, due to the lightweight design requirements of the helicopter, the gear system is thin-walled design. When the gear teeth are poorly lubricated or the lubricating oil film is unstable, the cooling capacity of the lubricating oil will be reduced, which directly leads to the heating deformation of the system, the reduction of transmission performance, and even the transmission system failure and helicopter crash.

To consider the influence of tooth surface roughness on lubrication in actual engineering, scholars proposed a hybrid lubrication model for the lubrication performance analysis of gears. According to the description of the surface topography of the friction pair, the models are divided into two categories, which include stochastic and the deterministic model. Christensen^1^ proposed a stochastic model for hydrodynamic lubrication analysis between one-dimensional rough surfaces. Patir^2^ proposed an average flow model, which divided the lubrication area into dry contact and fluid lubrication area. Chang^3,4^ proposed a thermoelastic lubrication mechanism for non-Newtonian fluids, and the effect of temperature on the lubricating oil was taken into account. Venner^5,6^ studied the steady-state lubrication of line contact using the actual measured rough surface. The results indicated that deformation of the rough peak under load could not be ignored in lubrication analysis. Ai^7,8^ solved the transient lubrication problem of the three-dimensional rough surface through the use of multi-grid and incomplete system method. Based on the research of theoretical calculations of EHL, some scholars had applied this theory to the field of gear transmission.

To resolve the lubrication analysis of gear transmission, Evans et al.^9–11^ measured the microscopic profile of the gear surface and studied the effect of surface microstructure on the gear lubricant film and pressure distribution. Due to the complexity of the meshing process of spiral bevel gears, fewer scholars employed the theory of hydrodynamic lubrication into the analysis of spiral bevel gear elastohydrodynamic. Simon^12,13^ analysed the influence of the adjustment of the processing parameters of the spiral bevel gear on its lubrication performance, and found that reasonable processing parameters could significantly enhance the bearing capacity of the elastic oil film of the gear. Pu and Wang^14–17^ examined the effects of various speed directions on the lubrication state, and discovered the analysis of the contact area friction coefficient as well as temperature field distribution. Pei^18,19^ predicted the wear of tooth surfaces for the spiral bevel gears, then proposed a transient mixed lubrication model and a two-dof-torsional dynamic model.

When the spiral bevel gear operated under high speed and heavy load, the mesh force might be very high near resonance compared with static condition^20–23^. Noted that the mesh force was an important parameter of lubrication analysis^24,25^. On the basis of Zaretsky model, Cao^26,27^ gave the contact fatigue analysis under different contact paths considering the mixed lubrication. Gan^28^ adopted thermal analysis finite element software to study the bulk and flash temperature of spiral bevel gears based on the mixed EHL model. Recently, Sun^29^ analyzed the impact of contact point migration on the results of EHL analysis.

The contact parameters (inclusive of contact force, relative speed, and radius of curvature, etc.) during the mesh of the spiral bevel gear, continually mutated with the change of the meshing position. Therefore, it is essential to note that lubrication analysis of spiral bevel gears must consider the time-varying features of the contact parameters.

In this work, the case where the minor axis of the ellipse is different from the direction of the entrainment speed of the lubricating oil is considered. Also, the case where the impact of pressure of the lubricating oil on the tooth surface lubrication under high speed and heavy load is considered. Based on the solution of point contact elastohydrodynamic lubrication model, a calculation model for the spiral bevel gear lubrication is established. Then the variation of the thickness and pressure of the lubricating oil film during the meshing of the spiral bevel gear is analyzed, and the effects of different working conditions and surface parameters on oil film thickness are also calculated.

## Simplified model for lubrication analysis of spiral bevel gears for engineering applications

### Simplified model of line contact and point contact hybrid lubrication analysis

The numerical solution of EHL is complex, and it costs a significant amount of time to analyze each meshing position. Dowson et al.^30^ proposed an empirical formula to simplify the calculation of the elastic lubrication film thickness. Masjedi et al.^31,32^ performed a large number of numerical calculations and fitted the calculation results into a mixed lubrication empirical formula suitable for engineering applications.

Based on these studies, expressions are derived to predict the central and minimum film thickness. The expressions also predict the asperity load ratio, which is the percentage of the load in contact (carried by the surface asperities). These formulas are summarized below, and the applicable parameter ranges are shown in Table 1.

**Table 1:** Parameter range for lubrication model.

	*W*	*U*	*G*	*κ*	*σ*	*V*
Minimum	1.5 × 10^−7^	1 × 10^−12^	2500	1	0	0.005
Maximum	2.5 × 10^−4^	1 × 10^−10^	7500	8	5 × 10^−5^	0.03

Line-contact EHL^31^ (L model):

1 $$ H_{c} = h_{c} /R = 2.691W^{ - 0.135} U^{0.705} G^{0.556} \left( {1 + 0.2\overline{\sigma }^{1.222} V^{0.223} W^{ - 0.229} U^{ - 0.748} G^{ - 0.842} } \right) $$

2 $$ H_{{{\text{min}}}} = h_{{{\text{min}}}} /R = 1.652W^{ - 0.077} U^{0.716} G^{0.695} \left( {1 + 0.026\overline{\sigma }^{1.120} V^{0.185} W^{ - 0.312} U^{ - 0.809} G^{ - 0.977} } \right) $$

3 $$ L_{a} = 0.005W^{ - 0.408} U^{ - 0.088} G^{0.103} \left[ {{\text{ln}}\left( {1 + 4470\overline{\sigma }^{6.015} V^{1.168} W^{0.485} U^{ - 3.741} G^{ - 2.898} } \right)} \right] $$

Point-contact EHL^32^ (P model):

4 $$ H_{{\text{c}}} = h_{{\text{c}}} /R = 3.672W^{{ - 0.045\kappa^{0.18} }} U^{{0.663\kappa^{0.025} }} G^{{0.502\kappa^{0.064} }} \left( {1 - 0.573e^{ - 0.74\kappa } } \right) \times \left( {1 + 0.025\overline{\sigma }^{1.248} V^{0.119} W^{ - 0.133} U^{ - 0.884} G^{ - 0.977} \kappa^{0.081} } \right) $$

5 $$ H_{{{\text{min}}}} = h_{{{\text{min}}}} /R = 1.637W^{{ - 0.09\kappa^{ - 0.15} }} U^{{0.711\kappa^{ - 0.023} }} G^{{0.65\kappa^{ - 0.045} }} \left( {1 - 0.974e^{ - 0.676\kappa } } \right) \times \left( {1 + 0.141\overline{\sigma }^{1.073} V^{0.149} W^{ - 0.044} U^{ - 0.828} G^{ - 0.945} \kappa^{ - 0.395} } \right) $$

6 $$ L_{a} = 10W^{ - 0.083} U^{0.143} G^{0.314} \left[ {{\text{ln}}\left( {1 + \overline{\sigma }^{4.689} V^{0.509} W^{ - 0.501} U^{ - 2.90} G^{ - 2.870} } \right)} \right] $$

### A modified model considering the angle between the direction of the entrainment velocity and the minor axis of the ellipse

**Figure 1:**
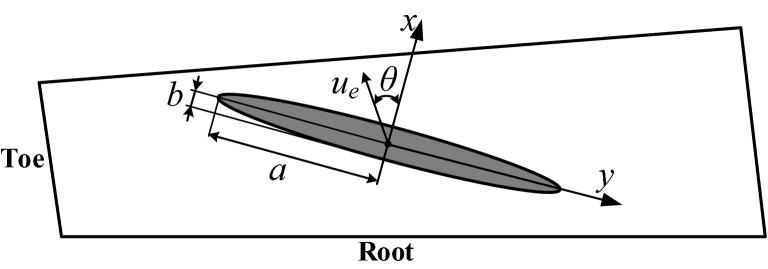
Schematic diagram of the angle *θ* between the lubricant entrainment and the minor axis of the contact ellipse.

In the EHL analysis of the tooth surface of the spiral bevel gear, the entrainment velocity of the meshing point is inconsistent with the minor-axis of the contact ellipse, and there is an included angle *θ* (Fig. 1).

It is necessary to consider the influence of the angle on the distribution of the lubricating oil film. Pu proposed a modified model (considering the angle *θ*), and the angle coefficient *ε* is calculated as follows^14^: (readers can refer to Ref.^14^ for more details.)

7 $$ \varepsilon = 1 + A_{1} \theta \left( {1 + B_{1} \overline{{U_{{\text{e}}} }}^{i} + C_{1} \overline{{P_{{\text{h}}} }}^{j} } \right) + A_{2} \theta^{2} \left( {1 + B_{2} \overline{{U_{{\text{e}}} }}^{m} + C_{2} \overline{{P_{{\text{h}}} }}^{n} } \right) + A_{3} \theta^{3} \left( {1 + B_{3} \overline{{U_{{\text{e}}} }}^{r} + C_{3} \overline{{P_{{\text{h}}} }}^{l} } \right) + A_{4} \theta^{4} $$

In the formula: $U_{e} = 12.7 \times u_{e} \times \eta_{0} /\left( {R_{x} E^{\prime } } \right)$, $P_{h} = p_{h} /E^{\prime }$.

The angle factor *ε* is applied to L model to obtain Line-contact EHL considering the influence of *θ* (LA model). Similarly, the PA model can be obtained.

Line-contact EHL considering the influence of *θ* (LA model):

8 $$ H_{{\text{c}}} = \varepsilon \left[ {2.691W^{ - 0.135} U^{0.705} G^{0.556} \left( {1 + 0.2\overline{\sigma }^{1.222} V^{0.223} W^{ - 0.229} U^{ - 0.748} G^{ - 0.842} } \right)} \right] $$

9 $$ H_{{{\text{min}}}} = \varepsilon \left[ {1.652W^{ - 0.077} U^{0.716} G^{0.695} \left( {1 + 0.026\overline{\sigma }^{1.120} V^{0.185} W^{ - 0.312} U^{ - 0.809} G^{ - 0.977} } \right)} \right] $$

10 $$ L_{a} = 0.005W^{ - 0.408} U^{ - 0.088} G^{0.103} \left[ {{\text{ln}}\left( {1 + 4470\overline{\sigma }^{6.015} V^{1.168} W^{0.485} U^{ - 3.741} G^{ - 2.898} } \right)} \right] $$

Point-contact EHL considering the influence of *θ* (PA model):

11 $$ H_{{\text{c}}} = \varepsilon \left[ {3.672W^{{ - 0.045\kappa^{0.18} }} U^{{0.663\kappa^{0.025} }} G^{{0.502\kappa^{0.064} }} \left( {1 - 0.573e^{ - 0.74\kappa } } \right) \times \left( {1 + 0.025\overline{\sigma }^{1.248} V^{0.119} W^{ - 0.133} U^{ - 0.884} G^{ - 0.977} \kappa^{0.081} } \right)} \right] $$

12 $$ H_{{{\text{min}}}} = \varepsilon \left[ {1.637W^{{ - 0.09\kappa^{ - 0.15} }} U^{{0.711\kappa^{ - 0.023} }} G^{{0.65\kappa^{ - 0.045} }} \left( {1 - 0.974e^{ - 0.676\kappa } } \right) \times \left( {1 + 0.141\overline{\sigma }^{1.073} V^{0.149} W^{ - 0.044} U^{ - 0.828} G^{ - 0.945} \kappa^{ - 0.395} } \right)} \right] $$

## Calculation of transient lubrication parameters of spiral bevel gears

Lubrication analysis of the spiral bevel gear requires parameters of each meshing point: the combined curvature of the contact surface-R_x_, R_y_, the contact load at the contact point-F, the size and direction of the entrainment velocity-${\varvec{v}}^{{\mathbf{e}}}$.

The tooth contact force *F* can be found through a loaded tooth contact analysis (LTCA) which can provide the contact force, contact path, transmission errors and bearing contact as a set of instantaneous contact ellipses^33^.

However, ${\varvec{v}}^{{\mathbf{e}}}$ and $\theta$ need to be calculated based on the kinematics of the contact points on the tooth. The contact points on the pinion and gear are represented by a pinion surface position vector ${\mathbf{r}}^{{\text{p}}}$ and a gear surface position vector ${\mathbf{r}}^{{\text{g}}}$. The pinion surface velocity vector ${\varvec{v}}^{{\text{p}}}$ and the gear surface velocity vector ${\varvec{v}}^{{\text{g}}}$ are defined in Eqs. (13) and (14). ${\varvec{\omega}}^{{\text{p}}}$ and ${\varvec{\omega}}^{{\text{g}}}$ are the angular velocity vectors of the pinion and gear respectively. The entraining velocity vector ${\varvec{v}}^{{\mathbf{e}}}$ is defined as per Eq. (16), and angle between the entraining velocity vector and the minor axis of the Hertzian ellipse $\theta$ can be derived from Eq. (17).

13 $$ {\varvec{v}}^{{\text{p}}} = {\varvec{\omega}}^{{\text{p}}} \times {\mathbf{r}}^{{\text{p}}} $$

14 $$ {\varvec{v}}^{{\text{g}}} = {\varvec{\omega}}^{{\text{g}}} \times {\mathbf{r}}^{{\text{g}}} $$

15 $$ {\varvec{v}}^{{\text{e}}} = \frac{{{\varvec{v}}^{{\text{g}}} + {\varvec{v}}^{{\text{p}}} }}{2} $$

16 $$ {\varvec{v}}_{{\text{t}}}^{{\text{e}}} = {\varvec{v}}^{{\text{e}}} - {\varvec{v}}^{{\text{e}}} \cdot {\mathbf{n}} \cdot {\mathbf{n}} $$

17 $$ \cos \theta = \frac{{{\varvec{v}}_{{\text{t}}}^{{\text{e}}} \cdot {\mathbf{b}}}}{{\left| {{\varvec{v}}_{{\text{t}}}^{{\text{e}}} } \right|\left| {\mathbf{b}} \right|}} $$

18 $$ p_{h} = \frac{3F}{{2\pi ab}} $$

**Figure 2:**
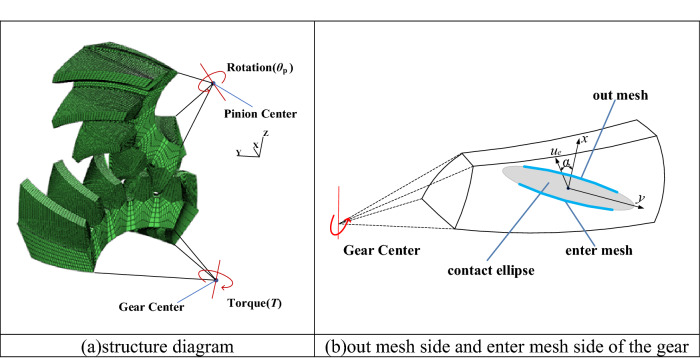
Structure diagram of spiral bevel gears.

Taking helicopter main transmission spiral bevel gears as an example, geometry parameters and the operating conditions of the spiral bevel gears are specified in Table 2. The structure diagram of the spiral bevel gears is shown in Fig. 2.

**Table 2:** Geometry and working parameters of the example of spiral bevel gear pair.

	Pinion	Gear
Number of teeth	27	35
Module (mm)	5.27	
Outer pitch diameter (mm)	142.29	184.45
Face width (mm)	36.5	
Pitch angle (°)	37° 39′	52° 21′
Face angle (°)	40° 7′	54° 38′
Root angle (°)	35° 22′	49° 15′
Outer cone distance (mm)	116.48	
Pressure angle at normal section (°)	20	
Mean helix angle (°)	40	
Shaft angle (°)	90	
Hand of spiral	Left hand	Right hand
ISO quality grade	4	4
Precision machining	Grinding	Grinding
Material	18CrNi4WA	18CrNi4WA
Surface hardness	HRC60	HRC60
Surface roughness(${\mu m}$)	Ra0.4	Ra0.4
Rotating speed(rpm)	6000	
Torque(Nm)		339

The main material parameters and lubricating oil characteristics of spiral bevel gears are summarized in Table 3.

**Table 3:** Material parameters and lubricating oil characteristics of spiral bevel gears.

Young's modulus of body, *E*_1_, *E*_2_ (Pa)	2.11 × 10^11^
Poisson's ratio of body, $v_{1}$, $v_{2}$	0.3
Surface roughness, ${\upsigma }$_1_, ${\upsigma }$_2_(m)	$4 \times 10^{ - 7}$
Vickers hardness *hd*_1_, *hd*_2_(HV)	697.5
Lubricant viscosity, $\eta_{0}$ (Pa.s)	0.04
Pressure viscosity coefficient, *α* (Pa^−1^)	2.2 × 10^−8^

Based on the results of LTCA, the contact parameters of the lubrication analysis of the spiral bevel gear are calculated. Table 4 shows the contact curvature, load and lubricant entraining velocity at different contact positions.

**Table 4:** Spiral bevel gear’s contact parameters of the meshing point.

*N*	θ_p_ (°)	*R*_*x*_ (mm)	*R*_*y*_ (mm)	*F* (N)	*u*_e_ (m/s)	$\theta$(°)	$p_{h}$(MPa)
1	− 10.285	12.61	770.79	211.91	27.61	55.81	613.44
2	− 9.2568	12.58	779.57	789.49	27.15	55.80	795.33
3	− 8.2282	12.55	788.57	1393.5	26.70	55.75	967.30
4	− 7.1996	12.51	797.85	2224.4	26.25	55.70	1137.98
5	− 6.171	12.47	807.40	3088.7	25.79	55.65	1274.41
6	− 5.1424	12.42	817.26	3994.1	25.34	55.59	1392.18
7	− 4.1138	12.37	827.43	4902.2	24.88	55.53	1493.86
8	− 3.0852	12.32	837.93	5775.2	24.43	55.47	1580.82
9	− 2.0566	12.25	848.79	6582.1	23.98	55.42	1654.30
10	− 1.028	12.19	860.03	7251.8	23.53	55.36	1711.40
11	0	12.12	871.63	7636.6	23.07	55.31	1743.03
12	1.0286	12.04	883.67	7095.7	22.62	55.24	1699.10
13	2.0609	11.96	896.09	6504.7	22.17	55.18	1648.83
14	3.0893	11.88	909.10	5679.8	21.72	55.12	1573.63
15	4.1179	11.78	922.61	4817.8	21.27	55.06	1487.42
16	5.1466	11.69	936.74	3909.9	20.83	55.01	1385.14
17	6.1751	11.58	951.46	2996.1	20.38	54.95	1264.95
18	7.2039	11.47	966.83	2115.2	19.93	54.90	1122.97
19	8.2323	11.36	982.90	1300.0	19.49	54.85	949.64
20	9.2609	11.24	999.69	625.55	19.04	54.80	739.04
21	10.29	11.11	1017.36	22.818	18.60	54.70	96.77

**Figure 3:**
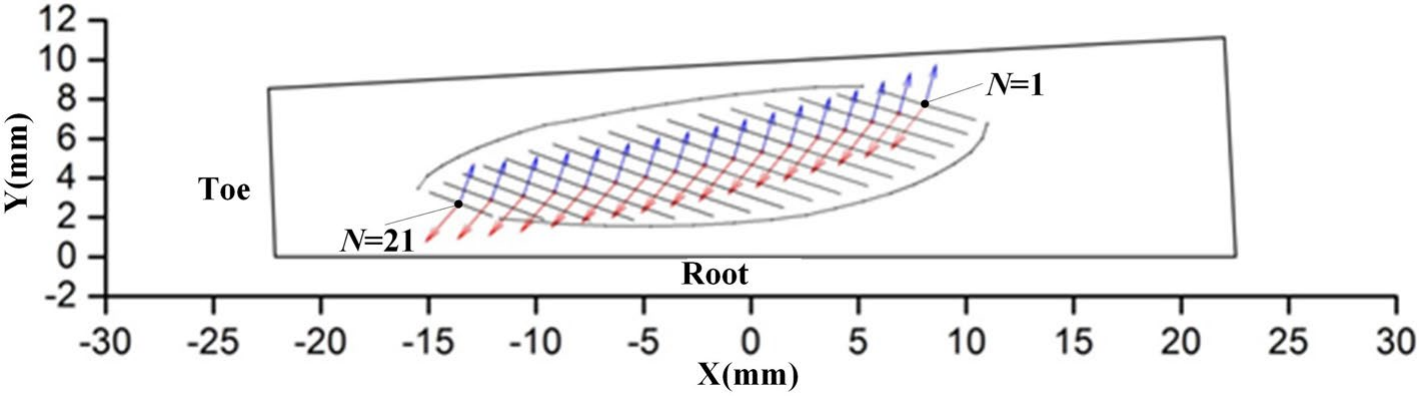
The lubricant entraining velocity and the minor axis of the contact ellipse of the different meshing positions of the gears.

According to the contact parameters of Table 4, the lubricant entraining velocity ${\mathbf{v}}^{{\mathbf{e}}}$ and the minor axis of the contact ellipse are obtained, as shown in Fig. 3. (The blue arrows are the direction of the minor axis of the ellipse, and red arrows are the direction of the entrainment velocity vector, respectively.)

**Figure 4:**
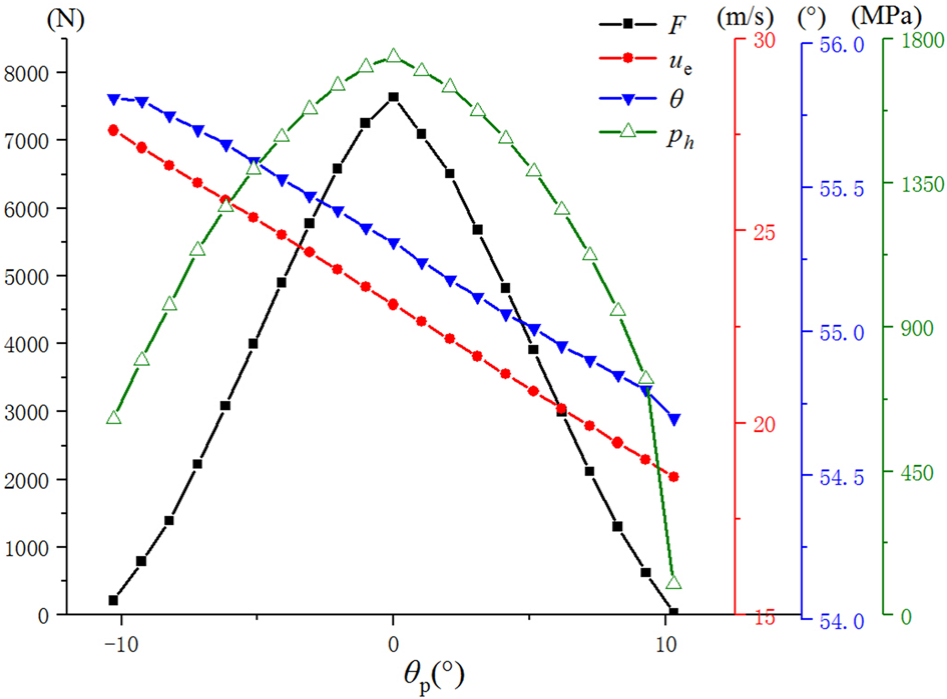
Contact load, lubricant entraining velocity and angle changing with the meshing position.

The changes of the contact load *F*, entraining velocity ${\varvec{v}}^{{\mathbf{e}}}$ and angle $\theta$ are shown in Fig. 4. The driven gear tooth surface enters the mesh from the tooth tip near the large end side, and is engaged from the tooth root near the small end side. Compare the different engagement positions, the entraining velocity at addendum (enter mesh) is maximal, and the entrainment at the root position (out mesh) is minimal. As the meshing position moves from the addendum to the root, the angle between the lubricant entraining velocity and the minor axis of the contact ellipse becomes small.

Figure 4 shows the meshing process, the contact force changes from 22.8 to 7636.6 N, the variation range being close to 100% and the maximum force appears in the contact center; the entrainment velocity changes from 18.6 to 27.6 m/s, the variation range reaches 32.6% and the maximum speed appears at the beginning of mesh; the angle $\theta$ changes from 54.7°to 55.8°, with a small variation (only 2%). This is due to the small change of tooth surface radian in the meshing process.

## Point contact numerical model and oil film thickness calculation

Based on the numerical calculation method of the circular contact EHL model provided by Venner^34^ (Moreover, this model had been validated well agreed with relevant friction experiments and readers can refer to research^35^ for more details.), the coordinate transformation method is used in the oil film distribution equation, and the angle between the entraining velocity and the minor axis of Hertzian ellipse is considered. This makes it suitable for the analysis of the lubrication characteristics of spiral bevel gears.

### Numerical solution method of lubrication equation

Using the coordinate transformation in the oil film thickness equation, the angle *θ* is placed in the calculation formula, and the Reynolds equation is:

19 $$ \frac{\partial }{\partial x}\left( {\frac{{\rho h^{3} }}{12\eta }\frac{\partial p}{{\partial x}}} \right) + \frac{\partial }{\partial y}\left( {\frac{{\rho h^{3} }}{12\eta }\frac{\partial p}{{\partial y}}} \right) = u_{e} \cos \theta \frac{{\partial \left( {\rho h} \right)}}{\partial x} + u_{e} \sin \theta \frac{{\partial \left( {\rho h} \right)}}{\partial y} + \frac{{\partial \left( {\rho h} \right)}}{\partial t} $$

After considering the angle *θ*, the Reynolds equation of the film thickness is unchanged, and the oil film thickness equation^34^ becomes:

20 $$ h = h_{0} + \frac{{\left( {x\cos \theta - y\sin \theta } \right)^{2} }}{{2R_{x} }} + \frac{{\left( {x\sin \theta + y\cos \theta } \right)^{2} }}{{2R_{y} }} + \frac{{1 + R_{x} /R_{y} }}{{2\pi \varepsilon_{1} }}\iint_{\Omega } {\frac{{p\left( {x^{\prime } ,y^{\prime } } \right)}}{{\sqrt {\left( {x - x^{\prime } } \right)^{2} + \left( {y - y^{\prime } } \right)^{2} } }}dx^{\prime } dy^{\prime } + \sigma_{1} \left( {x,y} \right) + \sigma_{2} \left( {x,y} \right)} $$

where *h*_0_ is a function of time and denotes the normal approach of the two rigid body surfaces, (*x*cos*θ*-*y*sin*θ*)^2^/2*R*_x_ and (*x*sin*θ*-*y*cos*θ*)^2^/2*R*_y_ are the transient geometry before elastic deformation, $\varepsilon_{1} = \mathop \smallint \limits_{0}^{\pi /2} \sqrt {1 - \left( {1 - \kappa^{2} } \right)\sin^{2} \xi } {\text{d}}\xi$, $\kappa = a/b$, Ω indicates the entire solution area. The solution area:− 4.5a ≤ x ≤ 1.5a, − 3.0b ≤ y ≤ 3.0b. (Material parameters and lubricating oil characteristics *E*, $v$, ${\upsigma }_{1}$, ${\upsigma }_{2}$, *hd*, $\eta_{0}$, *α* are enclosed in Table 3, contact parameter $R_{x}$, $R_{y}$, $\theta$, *u*_e_ are enclosed in Table 4.)

The method of changing the oil film thickness equation transforms the initial position of the contact surface so that the minor axis of Hertzian ellipse rotates along the coordinate system of the Reynolds equation.

### Analysis of transient lubrication performance of spiral bevel gear based on point contact numerical model

**Figure 5:**
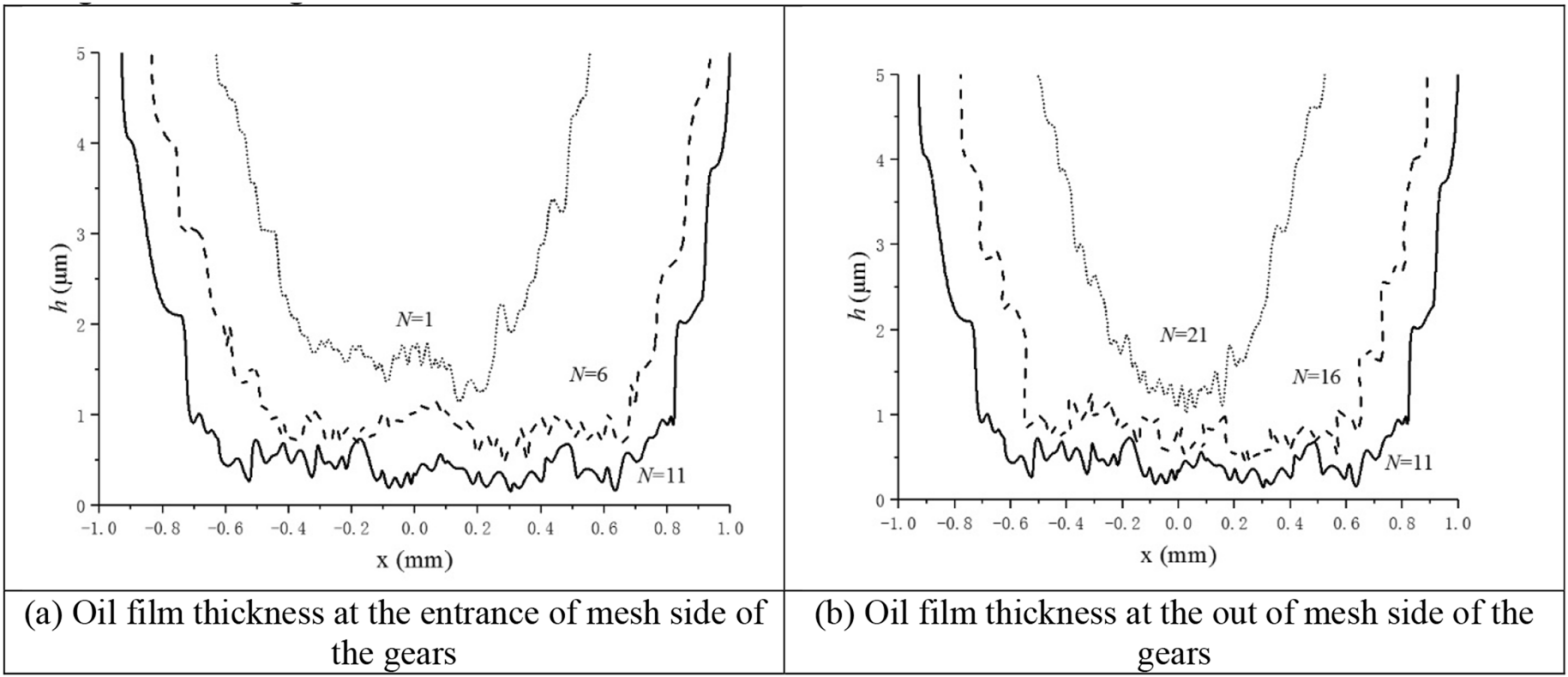
Oil film thickness distribution at different contact points.

According to the contact parameters of spiral bevel gear in Table 4, the point contact EHL model is used to analyze each contact point. Figure 5 shows oil film thickness of the meshing intermediate position (*N* = 11) is the smallest, 0.2 μm. While the oil film thickness at the enter mesh side (*N* = 1) and the out mesh side (*N* = 21) is larger, reaching 1.4 μm. During the meshing process of the spiral bevel gear, the change of the size and direction of the entrainment velocity of different contact points is small, but the load change of the contact point is obvious. This indicates that the contact point load is the main factor affecting the oil film thickness in the contact area.

A comparison of the oil film thickness at the enter mesh side and the out mesh side (Fig. 5) shows that the oil film thickness at the out mesh side is smaller than that at the enter mesh side in the case where the contact force is equal.

The oil film thickness of the driven gear at the root is smaller than that at the top of the driven gear. This results from both the decrease of the lubricant entraining velocity and the decrease of the entrainment angle $\theta$ at the out mesh side. It also shows that tooth surface roughness has a great influence on the distribution of oil film center.

**Figure 6:**
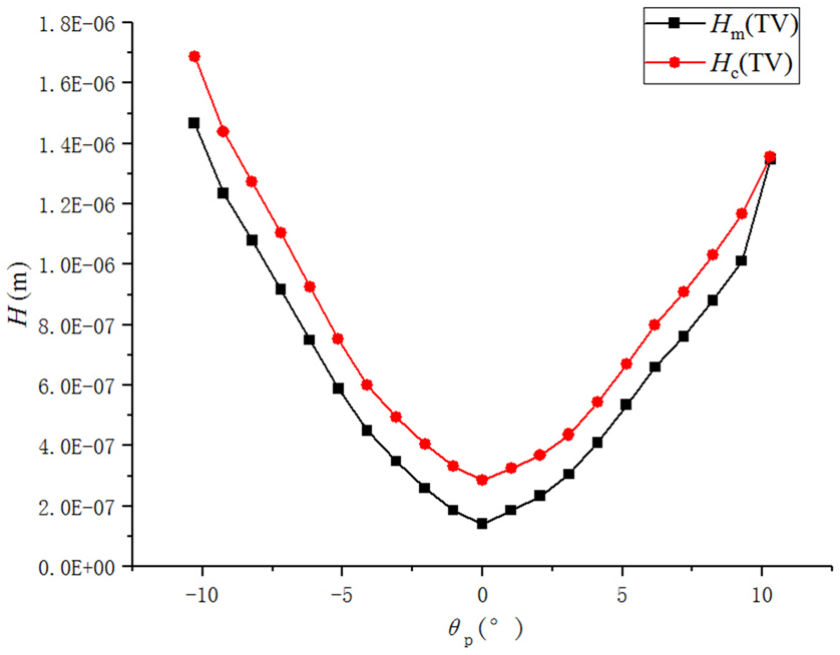
Oil film thickness at each mesh point calculated by the point contact numerical model.

The oil film thickness distribution at each contact point of the gear is shown in Fig. 6. During the gear meshing process, the distribution of oil film thickness at each contact point is clearly consistent with the load change trend. (light load- heavy load- light load). Under light load, the contact area is narrow, the thickness of the oil film is relatively large, and the oil film thickness at the out mesh side is significantly contracted. Under heavy load, the contact area is widened with the Hertz contact deformation occurring on the tooth surface, the oil film distribution in the contact area is relatively uniform, and the necking phenomenon at the out mesh side of oil film thickness still exists.

## Compare lubrication characteristics of different models

In the numerical solution of the point contact EHL model, the boundary conditions and initial values need to be changed with the contact parameters. In the analysis of the spiral bevel gear lubrication, the parameters of the contact point constantly change with the meshing position. It is necessary to amend the calculation parameters continuously to obtain the oil film thickness and pressure distribution, which will take considerable time. In this section, the simplified model of gear lubrication is used to analyze the lubrication of spiral bevel gears, and the calculation results of different models are compared.

### Comparison of lubrication analysis results of different simplified models

**Figure 7:**
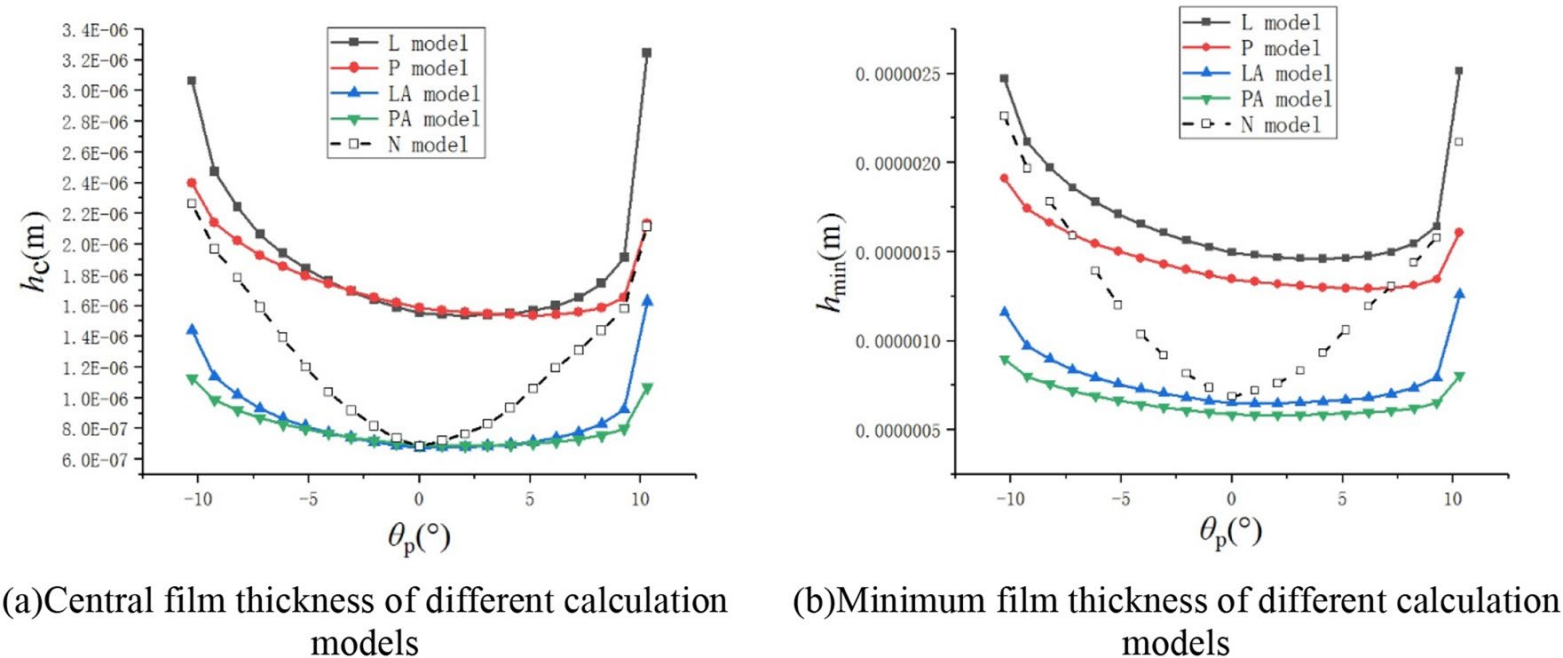
Central oil film thickness and minimum oil film thickness for different calculation models.

For contact lubrication of spiral bevel gears, the contact area ellipse is relatively large. Researchers^36^ believe that it is closer to line contact and can be solved using a line contact model. In order to compare the applicability of different models in the analysis of spiral bevel gear lubrication, the central film thickness (*h*_c_) and minimum film thickness (*h*_min_) calculated by different models are shown in Fig. 7.

It can be seen that the oil film thickness calculated by the PA model is close to that of LA model, and the calculation result of the PA model is smaller. The difference between the calculation results is whether the correction coefficient ($\varepsilon$) of the angle is considered or not. In this example, the correction coefficient *ε* of each meshing position is about 0.43 to 0.5. The calculation result of the correction coefficient *ε* is consistent with the calculation result^37^ of "effect of arbitrary entrainment angle in elastohydrodynamic lubrication elliptical and circular contacts".

In Fig. 7, the changing trends of line and point contact formulae are similar in the middle position and different in both sides. It is because that film thickness is very little influenced by load (in Eq. (1): − 0.135 exponent) and very much influenced by entrainment velocity (in Eq. (1): 0.705 exponent).

Using different formulae to calculate oil film thickness, the main difference is the calculation of force. $W = F/\left( {BE^{\prime } R} \right)$ in line contact and $W = F/\left( {E^{\prime } R^{2} } \right)$ in point contact. (*B* = 2*b*, choose long semi-axis length of Hertz contact ellipse to calculate contact length, the change of *b* is very small.) Although the force changes greatly, from 22 to 7636 N, the influence of force in the formula is small. Only when the force approaches 0 in both sides (22/7636≈0), film thickness of different formulae will be different.

Using L model to calculate film thickness of the spiral bevel gears will cause a large calculation error (Fig. 7). By comparing the calculation results of different simplified models and point contact lubrication numerical model (Table 5): it can be found that the calculation of the minimum film thickness by LA model is more accurate, and the prediction of the change tendency of the film thickness is more accurate. In conclusion, LA model is the closest model to the simulation results of the point contact numerical model (N model). For the lubrication analysis of spiral bevel gears, it is recommended to use the LA model rather than L model.

**Table 5:** Comparison of calculation results of different models with numerical simulation results.

	*h*_min_ (m)	*h*_c_ (m)
P model	5.02 × 10^−7^	6.53 × 10^−7^
L model	1.70 × 10^−6^	1.89 × 10^−6^
PA model	6.58 × 10^−7^	7.95 × 10^−7^
LA model	7.77 × 10^−7^	8.65 × 10^−7^

### Effect of working load on oil film thickness of spiral bevel gear

The load change in the tooth surface of the spiral bevel gear affects the contact area parameters. The gears in Table 4 are taken as an example, three working conditions in Table 6 are selected to analyze the effect of load on the oil film thickness at each contact point of the spiral bevel gear.

**Table 6:** working load of the spiral bevel gear.

*N*_w_	*P*_w_ (kW)	*T* (Nm) (6000 r/min)
1	213	339
2	378	602
3	450	716

**Figure 8:**
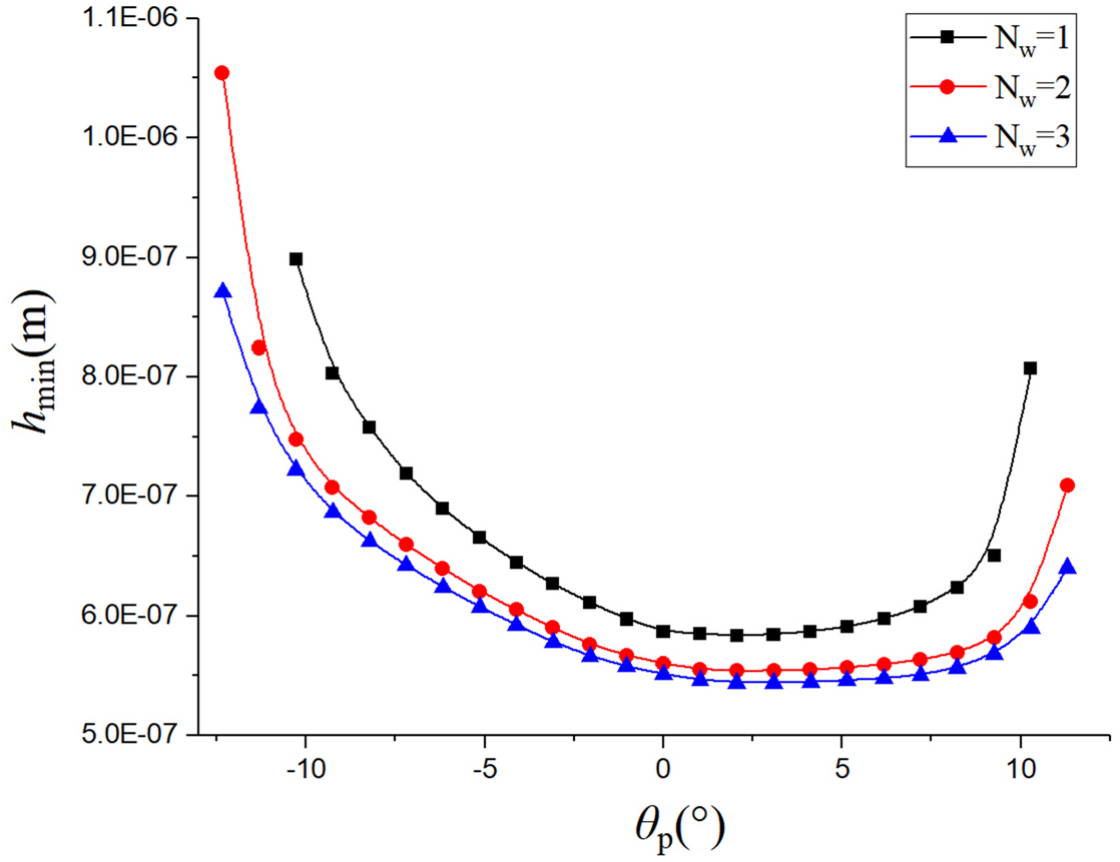
Oil film thickness under different working conditions.

In the simplified point contact model, the variations of the oil film thickness on the tooth surface under different working conditions are compared (Fig. 8). It can be seen that film thickness of the tooth surface decreases as the gear torque load increases. As the load increases, the contact area of the tooth surface becomes larger, and the range of the rotational angle of the gear also increases.

## Conclusion

In this paper, an engineering calculation model is introduced for point-contact EHL analysis of spiral bevel gears. Through numerical solution, the point contact EHL analysis under different loads and entraining velocity directions is performed. Using different simplified models and numerical models to calculate the oil film thickness and change trend of helicopter spiral bevel gears. The conclusions drawn from the present study include:

1. The instantaneous contact area of the spiral bevel gear is a long and narrow ellipse, and the influence of θ (the angle between the lubricant entraining velocity and the minor axis of the contact ellipse) should be considered in the EHL analysis of the spiral bevel gear.
2. During gear meshing process, the contact force *F* change range is close to 100%, contact force first increases and then decreases; the entrainment velocity ${\mathbf{v}}^{{\mathbf{e}}}$ reduced by 32.6%; the angle θ reduced by 2%. Due to the decrease of ${\mathbf{v}}^{{\mathbf{e}}}$ and angle θ, the film thickness at the out mesh side is smaller than that at the enter mesh side under the same contact force.
3. When the contact load is small, the contact area will be narrow, the film thickness will be relatively large, the film thickness will have a noticeable shrink at the out mesh side of the spiral bevel gears. When the contact load is high, the Hertz contact deformation will be evident, which leads to the enlargement of the contact area, the film thickness will be reduced and the distribution will become uniform.
4. The LA model is recommended for helicopter main transmission spiral bevel gears lubrication analysis, and the calculation result of the LA model is closer to that of the theoretical model. However, the simplified model’s film thickness distribution form is relatively gradual during the meshing process.
5. For helicopter main transmission spiral bevel gears, it is recommended that the tooth surface roughness reach Ra0.4 or better. The tooth surface roughness has a great influence on the distribution of oil film in the center. The stability of lubricating oil film can be effectively improved by reducing the surface roughness, especially the roughness on the meshing trace line. When the input condition of spiral bevel gears is determined, increasing the contact area of tooth surface can directly reduce the contact stress, increase film thickness and improve the lubrication stability.

## Appendix

### Notation

**Table:**

a	Semi-axis of the Hertzian contact ellipse in the x-direction, m
b	Semi-axis of the Hertzian contact ellipse in the y-direction, m
E_1_, E_2_	Young’s modulus of pinion and gear, respectively
$v_{1}$, $v_{2}$	Poisson’s ratios of pinion and gear, respectively
$E^{\prime}$	Effective Young's modulus,$1/E^{\prime } = 0.5\left[ {\left( {1 - v_{1}^{2} } \right)/E_{1} + \left( {1 - v_{2}^{2} } \right)/E_{2} } \right]$
h	Film thickness, m
$h_{{\text{c}}}$	Central film thickness, m
h_min_	Minimum film thickness, m
H	Dimensionless film thickness, H = h/R
H_c_	Dimensionless central film thickness, H_c_ = h_c_/R
H_min_	Dimensionless minimum film thickness, H_min_ = h_min_/R
$L_{a}$	Asperity load ratio (percentage)
x	x-coordinate, m
y	y-coordinate, m
R_x_	Effective radius in the x-direction, m
R_y_	Effective radius in the y-direction, m
R	Equivalent contact radius,1/R = (cos θ)^2^/R_x_ + (sin θ)^2^/R_y_, m
${\upsigma }$	Surface roughness, standard deviation of the surface heights, m
$\overline{\sigma }$	Dimensionless surface roughness,$\overline{\sigma } = \sigma /R$
F	Contact force at the contact point, N
B	Contact length, m
W	Dimensionless load parameter, $W = F/\left( {BE^{\prime } R} \right)$ in line contact and $W = F/\left( {E^{\prime } R^{2} } \right)$ in point contact
G	Dimensionless material number, G = $E^{\prime } \alpha$
hd	Vickers hardness, Pa
$V$	Dimensionless hardness number,$V = hd/E^{\prime }$
$\eta$	Lubricant viscosity, Pa.s
$\eta_{0}$	Lubricant viscosity at atmospheric pressure, Pa.s
U	Dimensionless speed number,$U = \left( {\eta_{0} u_{e} } \right)/\left( {E^{\prime } R} \right)$
$\kappa$	Hertzian contact ellipticity,$\kappa = a/b$
θ	angle between the entraining velocity vector and the minor axis of Hertzian ellipse
$\varepsilon$	Correction factor, represents the influence of θ
P	Dimensionless parameter of pressure,$P = p/p_{h}$
$\overline{\lambda }$	$\overline{\lambda } = \left( {12\eta_{0} u_{e} R_{x}^{2} } \right)/\left( {b^{3} p_{h} } \right)$
$\rho_{0}$	Lubricating oil density at normal atmospheric pressure
N	Number of the contact position
θ_p_	Rotation angle of gear
$p_{h}$(GPa)	Maximum Hertz contact stress,$p_{h} = \left( {3F} \right)/\left( {2\pi ab} \right)$
$\alpha$(Pa^−1^)	Pressure viscosity coefficient, α = 2.2 × 10^–8^ Pa^−1^
${\mathbf{r}}^{{\text{p}}}$	Pinion surface position vector
${\mathbf{r}}^{{\text{g}}}$	Gear surface position vector
${\varvec{\omega}}^{{\text{p}}}$	The angular velocity vectors of the pinion
${\varvec{\omega}}^{{\text{g}}}$	The angular velocity vectors of the gear
${\varvec{v}}^{{\text{p}}}$	Pinion surface velocity vector
${\varvec{v}}^{{\text{g}}}$	Gear surface velocity vector
${\varvec{v}}^{{\mathbf{e}}}$	Entraining velocity vector
${\varvec{v}}_{{\text{t}}}^{{\text{e}}}$	${\varvec{v}}^{{\mathbf{e}}}$ projection on the tangent plane
$u_{e}$	Entraining velocity, $u_{e} = \left| {{\mathbf{v}}_{{\text{t}}}^{{\text{e}}} } \right|$, m/s
P model	Point-contact EHL
L model	Line-contact EHL
PA model	Point-contact EHL considering the influence of θ
LA model	Line-contact EHL considering the influence of θ
N model	Point contact numerical model
N_w_	Working condition number
P_w_	Input power, kW
T	Input torque, Nm
